# Accuracy of prediction of simulated polygenic phenotypes and their underlying quantitative trait loci genotypes using real or imputed whole-genome markers in cattle

**DOI:** 10.1186/s12711-015-0179-4

**Published:** 2015-12-23

**Authors:** Saeed Hassani, Mahdi Saatchi, Rohan L. Fernando, Dorian J. Garrick

**Affiliations:** Department of Animal and Poultry Breeding and Genetics, Gorgan University of Agricultural Sciences and Natural Resources, Gorgan, Iran; Department of Animal Science, Iowa State University, Ames, 50011 USA

## Abstract

**Background:**

More accurate genomic predictions are expected when the effects of QTL (quantitative trait loci) are predicted from markers in close physical proximity to the QTL. The objective of this study was to quantify to what extent whole-genome methods using 50 K or imputed 770 K SNPs (single nucleotide polymorphisms) could predict single or multiple QTL genotypes based on SNPs in close proximity to those QTL.

**Methods:**

Phenotypes with a heritability of 1 were simulated for 2677 Hereford animals genotyped with the BovineSNP50 BeadChip. Genotypes for the high-density 770 K SNP panel were imputed using Beagle software. Various Bayesian regression methods were used to predict single QTL or a trait influenced by 42 such QTL. We quantified to what extent these predictions were based on SNPs in close proximity to the QTL by comparing whole-genome predictions to local predictions based on estimates of the effects of variable numbers of SNPs i.e. ±1, ±2, ±5, ±10, ±50 or ±100 that flanked the QTL.

**Results:**

Prediction accuracies based on local SNPs using whole-genome training for single QTL with the 50 K SNP panel and BayesC0 ranged from 0.49 (±1 SNP) to 0.75 (±100 SNPs). The minimum number of local SNPs for an accurate prediction is ±10 SNPs. Prediction accuracies that were based on local SNPs only were higher than those based on whole-genome SNPs for both 50 K and 770 K SNP panels. For the 770 K SNP panel, prediction accuracies were higher than 0.70 and varied little i.e. between 0.73 (±1 SNP) and 0.77 (±5 SNPs). For the summed 42 QTL, prediction accuracies were generally higher than for single QTL regardless of the number of local SNPs. For QTL with low minor allele frequency (MAF) compared to QTL with high MAF, prediction accuracies increased as the number of SNPs around the QTL increased.

**Conclusions:**

These results suggest that with both 50 K and imputed 770 K SNP genotypes the level of linkage disequilibrium is sufficient to predict single and multiple QTL. However, prediction accuracies are eroded through spuriously estimated effects of SNPs that are distant from the QTL. Prediction accuracies were higher with the 770 K than with the 50 K SNP panel.

## Background

There has been considerable focus on the identification of quantitative trait loci (QTL) and on strategies for including genomic information in selection programs as the amount of data on the bovine genome has increased [1]. Many research groups are striving to identify QTL accounting for variation in quantitative traits because of the resulting significant scientific and economic benefits. To date, only a few functionally significant mutations have been shown to control phenotypic variation and used commercially for cattle breeding [2]. Genome-wide linkage analysis is the traditional method to identify QTL and has been successful in mapping major QTL. However, the success of QTL mapping has been limited by: (1) the low heritability of most complex traits; (2) the low resolution of genome scans using microsatellite markers which were usually spaced at intervals of about 10 centiMorgans (cM) throughout the genome; and (3) the imprecise definition of phenotypes and inadequate experimental designs that limit the power to detect QTL [3]. The availability of high-density single nucleotide polymorphism (SNP) genotypes across the whole genome has enabled more accurate prediction of breeding values than conventional pedigree-based methods, as well as the mapping of QTL across the genome [4]. Current routine genomic evaluations of cattle populations are performed using selected genotypes that are obtained from the ~54,000 SNPs that are included in the BovineSNP50 or so-called 50 K array. However, high-density Affymetrix (648,874 SNPs) and Illumina (777,962 SNPs, referred to as the 770 K array) genotyping arrays are now available [5]. Using high-density SNP maps increases the probability of co-segregation between SNPs and quantitative trait nucleotides (QTN) [6]. Since both genomic predictions and genome-wide association studies exploit linkage disequilibrium (LD) between anonymous SNPs and QTN, increasing the density of SNP maps is likely to refine the findings from genome-wide population analyses [7–11].

Genomic selection exploits genomic information and can be based on (1) direct genomic values (DGV), which are estimated breeding values, from marker effects and genotypes only, or (2) on genomic enhanced breeding values (GEBV) that also combine information from phenotypes. The construction of the prediction equation and subsequent estimation of DGV or GEBV is termed “genomic prediction” and is the first step in genomic selection [12]. Although genomic prediction does not seek to explicitly detect the QTL that underlie the trait of interest, many of the models applied to predict DGV or GEBV can be used for QTL analysis. While genomic selection is currently producing results that lead to increased accuracy of selection at young ages, the ability to correctly determine the QTL that underlie traits of interest would further increase the ability to construct robust and accurate prediction equations. Thus, as the number of genotyped animals (with reliable phenotypes) increases, the ability to identify significant QTL should increase. The identification of QTL that explain small amounts of genetic variation can be improved by increasing the power of QTL studies [13]. Multiple methods and models have been proposed for implementing genomic selection. All methods have to overcome the so-called *p* > *n* problem, which is that the number of markers (*p*) is usually much larger than the number of phenotypic records (*n*). One approach to this problem is to use Bayesian inference that allows for an oversaturated model [14]. In a Bayesian approach, prior knowledge about the distribution of the effects of SNPs is assumed, i.e. that many of the SNPs are likely to have small individual effects and only a few will have large effects [15]. This results in shrinkage procedures in which the prior information is used to coerce negligible effects towards zero [16]. In BayesA, effects of SNPs are assumed to have different variances, which are modeled as a scaled inverse Chi square distribution [14]. The prior assumptions in BayesB [14] include SNPs that have zero effect with probability π, and the complement with probability (1 − π) following an inverse Chi square distribution, with *v* degrees of freedom and scale parameter *S*. Like BayesA, effects of SNPs are assumed to have different variances. In BayesC, the prior assumptions are similar to those of BayesB except that the effects of SNPs are assumed to have a common variance. It has been shown that BayesC is less sensitive to prior assumptions than BayesB [17]. The definition of the probability π assumed in BayesB and BayesC depends on the density of the SNP panel and the genetic architecture of the trait. Assuming π = 0 in BayesB is equivalent to BayesA, and in BayesC, it is referred to as BayesC0. BayesCπ assumes that the mixture probability π is not known but is estimated from the data by assuming a uniform prior distribution [17].

All these methods are expected to generate predictions that are highly correlated with the phenotype measured in the training population, particularly if the number of available SNPs greatly exceeds the number of animals and the number of QTL that contribute to the phenotype. Since BayesA and BayesC0 estimate the phenotype by using a linear combination that includes the effects of each SNP across the genome, there is no guarantee that SNPs that are physically close to the QTL will dominate the prediction even in models in which the phenotype is determined by a single QTL. Non-random association of alleles reflects LD and is important in fine-scale mapping of QTL [18]. BayesB and BayesC with values of π set to reflect the fact that most SNPs are assumed to have zero effect might, through variable selection, identify this small set of SNPs that are physically close to one or more QTL, provided the LD between SNP and QTL is high for some of the SNPs that are in close proximity to the QTL, and low for those that are more distant including those on other chromosomes. To optimally design marker panels, it is necessary to have information on the LD, however, for beef cattle, this information is limited, and among beef cattle breeds, the primary focus has been on the Angus breed [19]. The main objective of this study was to quantify, for some commonly used Bayesian regression techniques, to what extent SNPs from the 50 K SNP panel or the imputed 770 K SNPs and that are in close proximity of QTL can locally predict single or multiple QTL in Hereford beef cattle.

## Methods

### Genotype and phenotype data

Committee approvals for animal care and use were not required for this study since the data came from existing industry databases. A total of 2677 purebred American Hereford animals were genotyped with the BovineSNP50 BeadChip (Illumina, San Diego, CA, USA) at GeneSeek (Lincoln, NE, USA) providing results on 54,555 SNPs. The DNA of registered Hereford cattle was obtained from cryo-preserved semen or from hair samples provided by artificial insemination organizations or individual breeders. None of the American Hereford animals were genotyped with the 770 K panel, but we had access to SNP genotypes for 364 Irish Hereford cattle genotyped with Illumina BovineHD BeadChip. Marker genotypes for the 2677 American Hereford cattle were imputed using BEAGLE software [20] based on the 364 Irish Hereford cattle and resulted in 764,830 polymorphic SNP genotypes at real or imputed loci.

Simulated phenotypes with a heritability of 1 based on SNP genotypes (0, 1 and 2) were created for these animals and used as response variables. Phenotypes used to simulate a single QTL simply represented the vector of SNP genotypes at the locus chosen to represent the QTL since multiplying by a simulated QTL effect would simply scale the vector by a constant. For multiple QTL, the phenotype of each animal was simply defined as the sum of the 42 genome-wide QTL, which means that all the QTL had the same effect and that the contribution of each QTL to the genetic variance of the polygenic trait depended on its allele frequency relative to that of the other loci.

### Prediction strategies

In this study, every 1000th ordered SNP from the 50 K panel was chosen to represent a QTL. Among those, the 42 polymorphic autosomal SNPs that were located in mapped regions were used in the analysis. Two prediction strategies were followed: (1) estimating the effects of only the SNPs that flanked the QTL with variable numbers of SNPs i.e. ±1, ±2, ±5, ±10, ±50 or ±100 and predicting the QTL based on the effects of these SNPs only (local training and prediction); (2) training on the whole genome using all SNPs except those assumed to be QTL and then predicting the QTL based on the effects of only the SNPs that flanked the true QTL location with variable numbers of SNPs i.e. ±1, ±2, ±5, ±10, ±50 and ±100 (whole-genome training and local prediction).

### Statistical model

Bayesian methods including BayesA, BayesB [14], BayesC0, BayesC [21] and BayesCπ [17] were used to estimate the effects of SNPs for genomic prediction. The following model was fitted to the simulated phenotypes for training:

$$y_{i} = \, \mu \, + \, \varSigma_{j} z_{ij} \beta_{j} \delta_{j} + \, e_{i} ,$$

where *y*_*i*_ is the simulated phenotype on animal *i*, *μ* is the overall mean of *y*, *z*_*i*j_ is equal to 0, 1 or 2 depending on the SNP genotype at marker locus *j* in individual *i*, *β*_*j*_ is the allele substitution effect associated with marker *j*, *δ*_*j*_ is a 1 or 0 indicator variable for inclusion or exclusion of marker *j* in the model, and *e*_*i*_ is a residual. Parameter π was assumed for BayesB and BayesC to be 0 (BayesA, BayesC0), 0.95 (BayesB0.95, BayesC0.95) or 0.996 (BayesB0.996, BayesC0.996) for the 50 K analyses, corresponding to fitting non-zero effects for 50 K, 2500 or 200 SNPs per iteration, whereas π was equal to 0 (BayesC0) or 0.9997 (BayesB0.9997, BayesC0.9997), corresponding to fitting 770 K or about 200 SNPs per iteration for 770 K SNP panels. High values of π were chosen to provide variable selection by assuming that most loci had zero effect while still ensuring that the number of SNPs fitted in each iteration was larger than the actual number of QTL (1 or 42) that contributed to the trait. In real data analyses, the number of QTL is not known, and BayesCπ in which π is estimated from the data is a practical alternative that was also used in this study. Markov chain Monte Carlo (MCMC) methods with 41,000 or 21,000 iterations for the 50 or 770 K SNP panels, respectively, were used to provide posterior mean estimates of effects of SNPs after discarding the first 1000 samples for burn-in. A smaller number of iterations was chosen for the 770 K analyses to reduce computing effort since preliminary analyses demonstrated rapid convergence of these models for both the simulated monogenic or polygenic phenotypic traits with a heritability of 1. Relationships between minor allele frequency (MAF) and prediction accuracies were also studied. All analyses were performed using GenSel software [22].

### Cross-validation

Animals were clustered into six groups using K-means clustering on pedigree estimates of additive genetic relationships between animals in order to increase within-group and decrease between-group relationships [23]. All six combinations of five groups were used for model training, with cross-validation performed by predicting each group not used in training. Cross-validation was only performed for the summed 42 QTL and included analyses undertaken using BayesCπ with starting values of π = 0.95 (BayesCπ0.95) or π = 0.996 (BayesCπ0.996) to compare accuracies in situations for which neither prior knowledge on the genetic architecture nor the likely values of the proportion or number of loci with zero effects were known.

## Results and discussion

### Convergence of the predictive ability of the Markov chain

A frequent criticism of MCMC-based Bayesian regression models is that convergence may not be achieved with a given number of iterations in the Markov chain. It was assumed that the single-QTL analyses with a heritability of 1 would converge faster than the analyses using the summed 42 QTL to represent a polygenic trait. A preliminary analysis using the simulated polygenic trait showed convergence of the chains with far less iterations than were performed in our study. Post-burn-in samples of SNP effects were averaged to obtain posterior means of SNP effects as the length of the chain increased. Using BayesC with π = 0.996 for 50 K SNPs, the correlation between phenotype and whole-genome prediction exceeded 0.97 at burn-in, 0.980 after 280 post-burn-in iterations, 0.983 after 6280 post-burn-in iterations, and then it failed to increase further. For a trait with a heritability of 1, this correlation represents the correlation between true and estimated genetic merit. Using BayesC with π = 0.9997 for 770 K SNPs, the correlation exceeded 0.99 at burn-in, and asymptoted to 0.996 within a further 300 iterations.

### Single QTL: local training and predictions

**Fig. 1:**
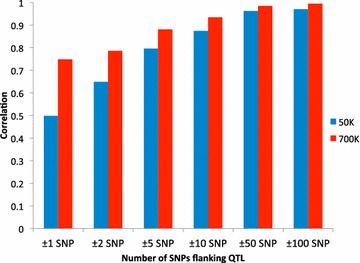
Accuracies for local training and predictions averaged over 42 QTL predicted using 50 or 770 K SNP panels and BayesC0

If the QTL locations are known (e.g. from genome-wide association studies or from the location of candidate genes), higher accuracy might be achieved by using increased SNP density in those regions and eliminating SNPs in other unassociated regions. To quantify this effect, local training and predictions were performed. Figure 1 shows accuracies (correlations between *y* and DGV) averaged over 42 QTL for local (±1 SNP, ±2 SNPs, ±5 SNPs, ±10 SNPs, ±50 SNPs and ±100 SNPs flanking the QTL) training and predictions with the 50 or 770 K SNP panels using BayesC0. Prediction accuracies ranged from 0.50 (±1 SNP) to 0.97 (±100 SNPs) and 0.75 (±1 SNP) to 0.99 (±100 SNPs) for the 50 and 770 K SNP panels, respectively. Accuracies increased as the numbers of SNPs flanking the QTL increased for both 50 and 770 K SNP panels. Regardless of the number of SNPs flanking the QTL, accuracies that were estimated with the 770 K panel were higher than with the 50 K panel but differences between these accuracies decreased as the number of SNPs flanking the QTL increased.

### Single QTL: whole-genome training and local predictions

**Fig. 2:**
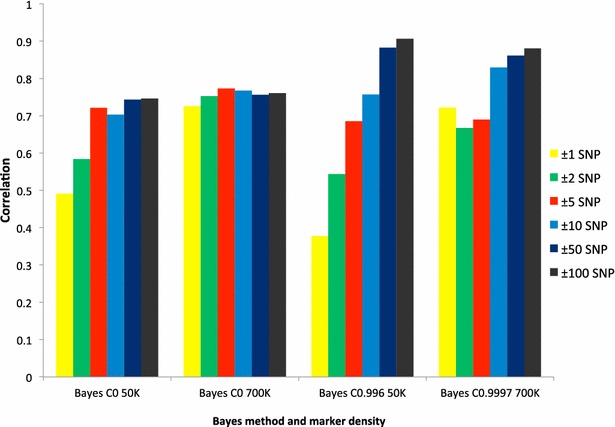
Accuracies for whole-genome training and local predictions averaged over 42 QTL predicted using the 50 or 770 K SNP panels and BayesC with different values of π (0, 0.996 or 0.9997)

Figure 2 shows the accuracies (correlations between *y* and DGV) averaged over 42 QTL for whole-genome training and local predictions that were estimated with the 50 and 770 K SNP panels using BayesC. Accuracies obtained with the 50 K SNP panel and BayesC0 were lowest and highest for ±1 SNP (0.49) and ±100 SNPs (0.75) flanking the QTL, respectively, with minor variations in accuracy for numbers of flanking SNPs between ±5 and ±100 SNPs. With the same 50 K SNP panel and BayesC0.996, accuracies increased steadily as the number of SNPs flanking the QTL increased from 0.38 (±1 SNP) and to 0.91 (±100 SNPs). Comparison of results obtained with BayesC0 and BayesC0.996 indicated that except for ±1, ±2 and, ±5 flanking SNPs, accuracies were higher for BayesC0.996. With the 770 K SNP panel and BayesC0, accuracies were higher than 0.70 for all numbers of flanking SNPs, with minor variations that ranged from 0.73 (±1 SNP) to 0.77 (±5 SNPs). With the 770 K SNP panel and BayesC0.9997, accuracies were lowest and highest for ±2 (0.67) and ±100 flanking SNPs (0.88), respectively. In this case, at least ±10 flanking SNPs are required for the accuracy to be higher than 0.80. With BayesC0, accuracies were higher with the 770 K than with the 50 K SNP panel regardless of the number of SNPs flanking the QTL but if π was not equal to 0, accuracies were lower with the 770 K than with the 50 K panel for ±50 and ±100 flanking SNPs.

Accuracies of whole-genome prediction for both SNP panels regardless of the number of flanking SNPs did not exceed 0.76. In contrast, accuracies for local prediction with ±100 flanking SNPs were close to 1. Estimating higher accuracies for local training than for whole-genome training may be due to the spurious interference of more distant SNPs in whole-genome training. Comparison of results from local and whole-genome training (Figs. 1, 2) indicates that accuracies of predictions from local training were higher than for whole-genome training for both 50 and 770 K SNP panels but the differences between the two prediction accuracies decreased as the number of SNPs flanking the QTL increased.

In general, the amount of LD between any two SNPs decreases as the physical distance between them increases. However, forces such as selection can cause markers that are far apart physically (or even on different chromosomes) to be in high LD with one another [24]. Generally, our results indicated that for both 50 and 770 K SNP panels, at least ±10 flanking SNPs were required to reach a reasonable accuracy. Increasing the number of SNPs used for genomic evaluation is expected to increase the accuracy of evaluations through better tracking of QTL [25]. However, previous studies on simulations showed conflicting results i.e. increasing SNP density to more than 50,000 (50 K) resulted in either no gain in evaluation reliability [26], very small gains [27], or large gains [8]. A study on real data showed that the use of high-density genotypes for 384 Norwegian Red bulls increased the correlations between predicted and observed reliabilities for milk yield, protein yield, and one measure of mastitis by 7 to 9 % whereas for four other traits (non-return rate at 56 days, days from calving to first insemination, somatic cell score, and another measure of mastitis) no or little increase was found [28]. Benefits from high-density genotypes may be small if most of the genetic variation is due to many QTL with very small effects [29]. Imputation losses can also affect reliability of evaluation if the number of animals with high-density genotypes is insufficient [30]. The number of flanking SNPs that are needed to detect a QTL depends on the distance over which LD extends. Increasing SNP density will increase the power to detect QTL and, to some extent, increase the precision of mapping. However, if LD is high across a chromosome segment, increasing SNP density may still not make it possible to map the QTL precisely within this segment. In such a case, each QTL could be tracked by many SNPs because no individual SNP is in complete LD with the QTL, especially in domestic animals in which LD extends across a large distance and SNP density is not high [31]. Generally, with high-density chips, the number of SNPs that are in high LD with the QTL will increase but the level of co-linearity between informative and uninformative SNPs will also increase as the number of SNPs that statistically account for phenotype error increase [26]. Several major factors that influence the accuracy of genomic selection have been reported in the literature: (1) LD extent between SNPs and QTL; (2) size of the training population (i.e. individuals that are both phenotyped and genotyped and used to construct the statistical models and predict the effects of SNPs); (3) heritability or genetic basis of the trait analyzed, and (4) distribution of QTL effects [24]. Accuracies of genomic prediction increased with BayesC when the number of flanking SNPs was equal to ±10 or more as π increased for both 50 and 770 K SNP panels. Generally, our results indicated that as far as accuracy of QTL prediction is concerned, the finite locus local prediction model performed better than the whole-genome infinitesimal model. Saatchi et al. [32] in a study on Hereford beef cattle showed that, with BayesC, accuracies of genomic predictions increased as π increased from 0 to 0.95 and 0.99 (fewer fitted markers in the model) for almost all studied traits. When π is small, we assume that the trait is likely affected by many QTL with small effects, and when π is large, a few large QTL are expected to influence the trait [17].

### Multiple QTL: whole-genome training and local predictions

**Fig. 3:**
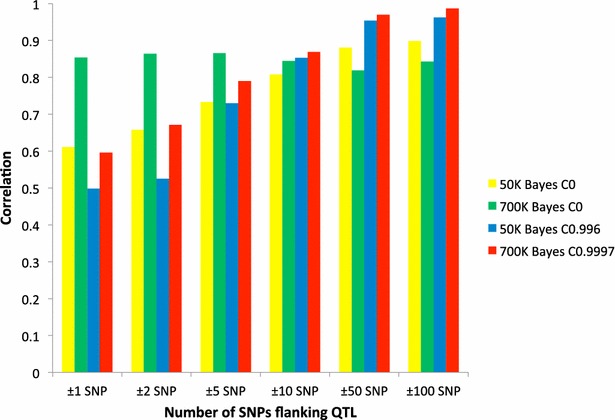
Accuracies for whole-genome training and local predictions in training data for a complex trait defined as the sum of 42 QTL predicted with 50 or 770 K SNP panels using BayesC with different values of π

Since most complex quantitative traits are associated with multiple QTL rather than a single QTL, the same 42 QTL used for single-QTL prediction were summed to represent a polygenic trait and whole-genome training and local (±1, ±2, ±5, ±10, ±50 and ±100 SNPs flanking the QTL) predictions were done with the 50 or 770 K SNP panels using BayesC (Fig. 3). Similar trends for multiple QTL prediction were found as previously reported for single QTL prediction but accuracies for multiple QTL prediction were generally higher than those obtained for a single QTL regardless of the number of flanking SNPs except for a few cases. Estimating higher prediction accuracies for multiple QTL may be due to higher LD for the summed 42 QTL than for a single QTL. In other words, local markers for one QTL may be in LD with other QTL even on another chromosome. Generally, multiple QTL are expected to reflect the underlying genetic architecture of complex traits better than single QTL. Meuwissen [33] concluded that multipoint-QTL mapping is expected to reflect the underlying genetic model better than single-QTL mapping. In particular, MCMC multi-QTL mapping approaches result in sharper QTL peaks because they estimate the effect of a QTL at the putative position conditional on all other QTL that are fitted in the model, whereas single-QTL mapping estimates the effect of a QTL at the putative position without accounting for effects of QTL at other positions, and thus, can detect an apparent effect due to LD with a large QTL at some other location.

### Cross-validation

**Fig. 4:**
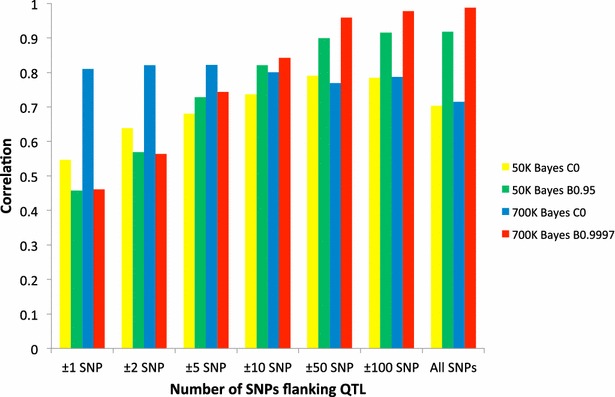
Accuracies for whole-genome training and local predictions averaged over cross-validation folds for a complex trait defined as the sum of 42 QTL predicted with 50 or 770 K SNP panels using BayesB or BayesC with different values of π

**Fig. 5:**
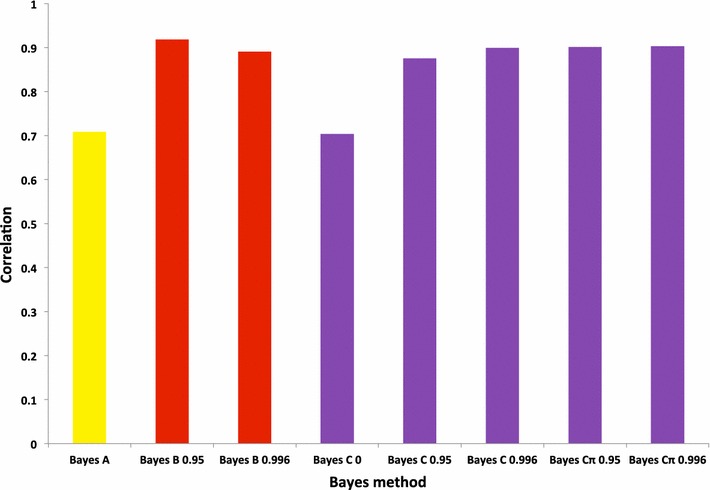
Average cross-validation accuracies of all validation groups for a complex trait defined as the sum of 42 QTL predicted with all 50 K SNPs excluding QTL by different Bayes methods and for π values

Figure 4 shows average cross-validation accuracies of whole-genome training and local predictions of all validation groups for the summed 42 QTL using 50 or 770 K SNP panels with BayesB or BayesC. For the 50 K SNP panel, accuracies ranged from 0.55 (±1 SNP) to 0.79 (±50 SNPs) and from 0.46 (±1 SNP) to 0.92 (all SNPs) for BayesC0 and BayesB0.95, respectively. For the 770 K SNP panel, accuracies ranged from 0.71 (all SNPs) to 0.82 (±5 SNPs) and from 0.46 (±1 SNP) to 0.99 (all SNPs) for BayesC0 and BayesB0.9997, respectively. Accuracies for the 770 K were higher than those for the 50 K SNP panel regardless of the number of flanking SNPs using either BayesB or BayesC. Increasing the number of SNPs flanking QTL did not change accuracies significantly for the 770 K SNP panel with BayesC0, whereas with BayesB0.95 and BayesB0.9997, accuracies increased. This clearly indicates that BayesB gives more emphasis to informative SNPs that are near the QTL than does BayesC as a result of its ability to provide greater shrinkage to SNPs with small effects since these SNPs have larger variance ratios than large effect markers. Although all the models estimate the phenotypes in the training data, they may vary in their accuracy for out-of-sample prediction, which is why cross-validation is necessary. Out-of-sample prediction is more difficult, because LD relationships may differ between testing and validation samples, or because models with many parameters can overfit the data to reflect their vagaries [34]. Figure 5 shows, for different Bayesian methods and π values, the average cross-validation accuracies of all validation groups for the summed 42 QTL using all 50 K SNPs excluding SNPs that were QTL. Accuracies ranged from 0.70 for BayesA and BayesC0 to 0.92 for BayesB0.95. Generally, the results indicated that Bayesian methods with π values higher than 0 resulted in higher accuracies but with π set to the same values, BayesB and BayesC did not differ in accuracy. The study of Wang et al. [35] based on the 15th QTL-MAS Workshop dataset concluded that BayesA, BayesB, BayesCπ performed similarly and satisfactorily for the estimation of genomic breeding values.

### Minor allele frequency

**Fig. 6:**
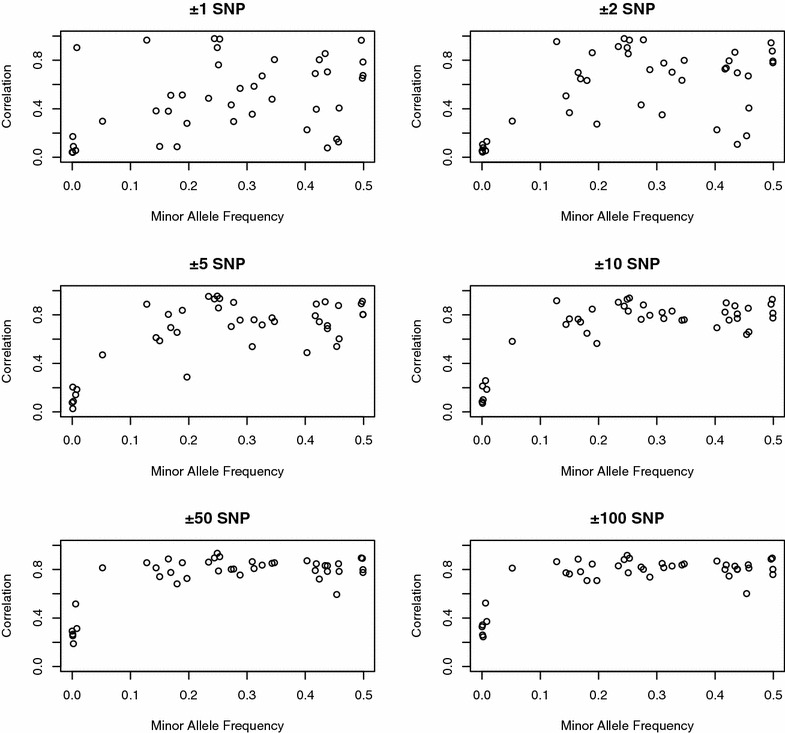
Accuracies for whole-genome training and local predictions obtained using BayesC0 with a 50 K SNP panel for 42 individual QTL plotted against the QTL minor allele frequencies

**Fig. 7:**
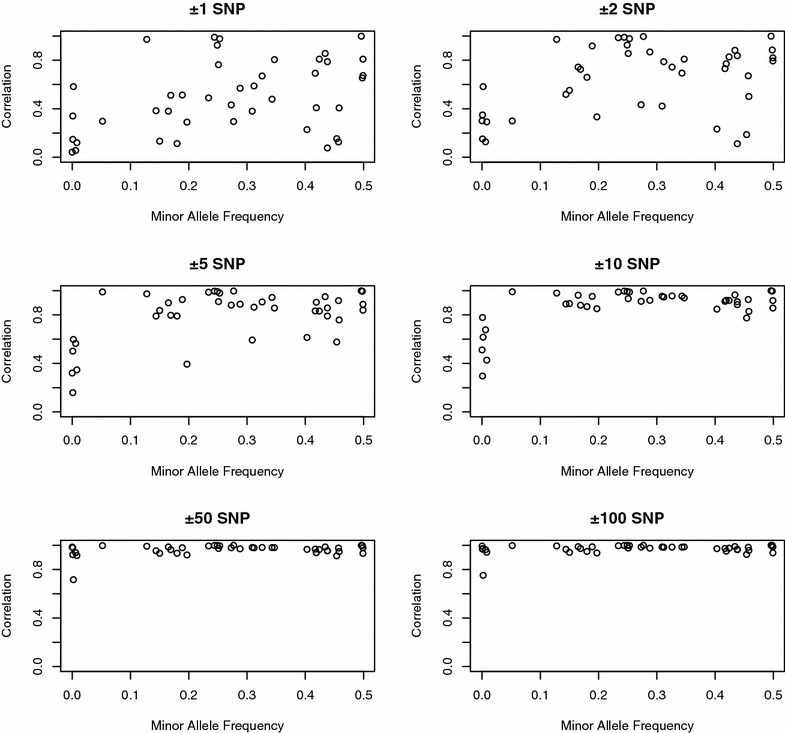
Accuracies for local training and predictions obtained using BayesC0 with a 50 K SNP panel for 42 individual QTL plotted against the QTL minor allele frequencies

MAF for the 42 QTL ranged from 0.0001 to 0.499 with an average of 0.27. Figure 6 shows whole-genome training and local prediction accuracies for the 42 QTL obtained with BayesC0 and the 50 K SNP panel plotted against their MAF. Overall, regardless of the number of flanking SNPs, accuracy of low MAF QTL is lower than that of QTL with a MAF higher than 0.15. Simple correlations between MAF and accuracies ranged from 0.37 to 0.67 for ±1 and ±10 flanking SNPs, respectively. Figure 7 shows local training and local predictions accuracies for the MAF of the 42 QTL obtained by BayesC0 for the 50 K SNP panel. As for whole-genome training, there was a positive correlation between MAF, and accuracies for local training ranged from 0.20 to 0.49 for ±10 and ±100 flanking SNPs, respectively, but fluctuated less for ±50 and ±100 flanking SNPs. Figure 7 shows that QTL with a low MAF had lower accuracies in the first four graphs but for the last two (±50 and ±100 flanking SNPs), these QTL showed high accuracies. In other words, for rare QTL with low MAF, a larger number of flanking SNPs is required in order to increase prediction accuracies. Using simulated data, Pérez-Encisco et al. [36] demonstrated that including all SNPs that are located within causal genes in the prediction model could dramatically increase prediction accuracy by ~40 % compared to using the SNPs that typically compose a genotyping array. Du et al. [37] in a study on LD in pig populations found that expected local LD increased with increasing MAF under the assumption that the MAF at two loci are independently and uniformly distributed.

### Selection

The genotypes used in this simulation represented actual genotypes for a real cattle population, but it is likely that the distribution of the allele frequencies of those genotypes will not reflect the distribution of allele frequencies of real QTN, because SNPs that are selected for genotyping arrays are chosen to be particularly informative and therefore tend to include more SNPs with a higher allelic frequency than those included in exonic variants [38]. Furthermore, for most traits of interest for genomic selection, artificial and perhaps also natural selection might have created long-range LD through the so-called Bulmer effect [39]. Studying the effects of such selection would require more complex simulations than those reported here, but they would likely reduce the ability to detect, in whole-genome analyses, SNPs with large effects near the QTL. Moreover, the simulation in our study assumed simulated traits with a heritability of 1, whereas in practice, only a few traits of interest in selection programs have a heritability higher than 0.5, and many have a heritability lower than 0.25. In such circumstances, many more genotyped animals would be required to achieve the accuracies reported in this study. Finally, most productive traits are likely to be influenced by many more QTL than the 42 that were simulated in this study, and many of these may have small effects, which would make them more difficult to detect than was the case in this study where all QTL had the same effect.

## Conclusions

This study applied single- and multiple-QTL predictions using SNPs that flank the QTL to American Hereford beef cattle genotypes and simulated phenotypes. Overall, our results suggest that for a dataset with less than 3000 animals, the LD of the 50 K SNP panel is sufficient to predict single or multiple QTL. Training on SNPs that are located close to the QTL rather than whole-genome SNPs resulted in higher prediction accuracies than whole-genome analyses, which indicates that predictive accuracy can be improved if prior knowledge of the QTL locations or of the likely informative SNPs was available. In our simulation, we assumed a complex trait that was influenced by only 42 QTL with a heritability of 1, which suggests that for traits with more QTL or lower heritabilities, predictions would be less accurate. As far as marker density is concerned, the 770 K SNP panel performed slightly better than the 50 K SNP panel for prediction of single and multiple QTL. Analysis of the QTL MAF shows that in order to get high prediction accuracies for rare QTL, a larger number of SNPs flanking the QTL (±50 or ±100 SNPs) is required. Bayesian regression methods that involve variable selection were better able to detect and localize QTL than methods that fitted all whole-genome SNPs simultaneously.
